# Supplementary material for: Additive global cerebral blood flow normalization in arterial spin labeling perfusion imaging
Source: PeerJ. 2015 Mar 17;3:e834. doi: 10.7717/peerj.834 (PMC4369335; doi:10.7717/peerj.834)
Supplement: Supplemental Information 3 — This SPM analysis used images that were not corrected for global cerebral blood flow. The first page has the table with the clusters of activation. Pages 2–7 show the 2 significant clusters of activation with crosshairs at the top 3 peaks of activation for each cluster. The last page has the table with the clusters of deactivation. There were no significant areas of deactivation. [file peerj-03-834-s003.pdf]

check increases UNSHIFT 20 subs, preLD, pbo day

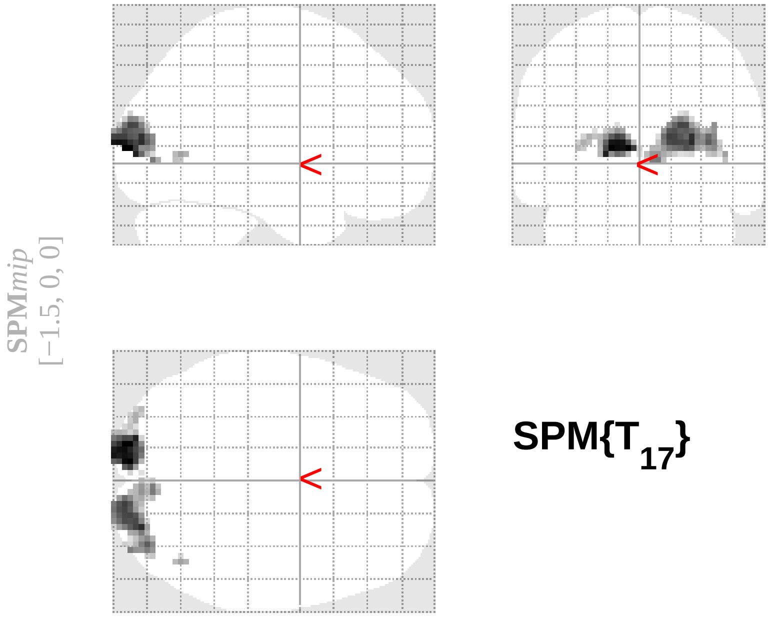

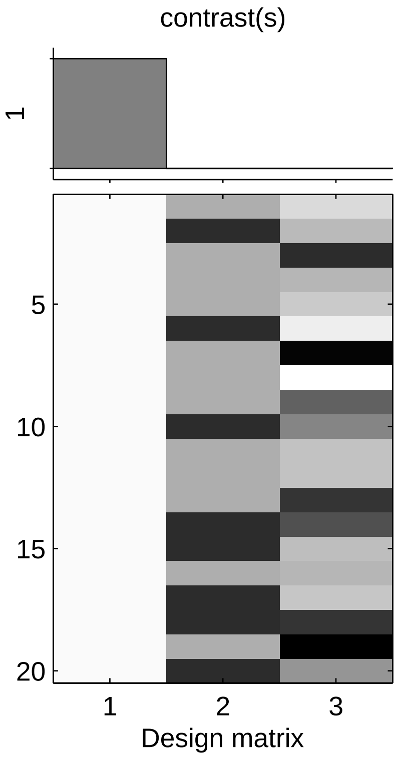

SPM{T_{17}}

SPMresults: .unshifted_pbo_preLD_all_subs
Height threshold T = 3.645767 {p<0.001 (unc.)}
Extent threshold k = 0 voxels

Statistics: ***p−values adjusted for search volume***

set−level		cluster−level				peak−level					mm mm mm
p	c	$p_{FWE-corr}$	$q_{FDR-corr}$	k_E	p_{uncorr}	$p_{FWE-corr}$	$q_{FDR-corr}$	T	$(Z_{\equiv})$	p_{uncorr}	
**0.986**	**3**	**0.002**	**0.000**	**119**	**0.000**	**0.178**	**0.174**	**6.13**	**4.40**	**0.000**	**−8 −93 6**
						0.180	0.174	6.12	4.39	0.000	−16 −93 6
						0.972	0.482	4.20	3.43	0.000	−28 −90 12
		**0.000**	**0.000**	**247**	**0.000**	**0.354**	**0.188**	**5.59**	**4.16**	**0.000**	**26 −87 9**
						0.451	0.188	5.39	4.06	0.000	14 −96 12
						0.476	0.188	5.34	4.04	0.000	20 −93 18
		**0.926**	**0.325**	**6**	**0.325**	**0.950**	**0.436**	**4.32**	**3.50**	**0.000**	**44 −66 3**

table shows 3 local maxima more than 8.0mm apart

Height threshold: T = 3.65, p = 0.001 (1.000)
Extent threshold: k = 0 voxels
Expected voxels per cluster, <k> = 6.691
Expected number of clusters, <c> = 8.00
FWEp: 7.013, FDRp: Inf, FWEc: 119, FDRc: 119

Degrees of freedom = [1.0, 17.0]
FWHM = 12.7 13.8 13.7 mm mm mm; 4.2 4.6 4.6 {voxels}
Volume: 1294110 = 47930 voxels = 486.0 resels
Voxel size: 3.0 3.0 3.0 mm mm mm; (resel = 88.62 voxels)

check increases UNSHIFT 20 subs, preLD, pbo day

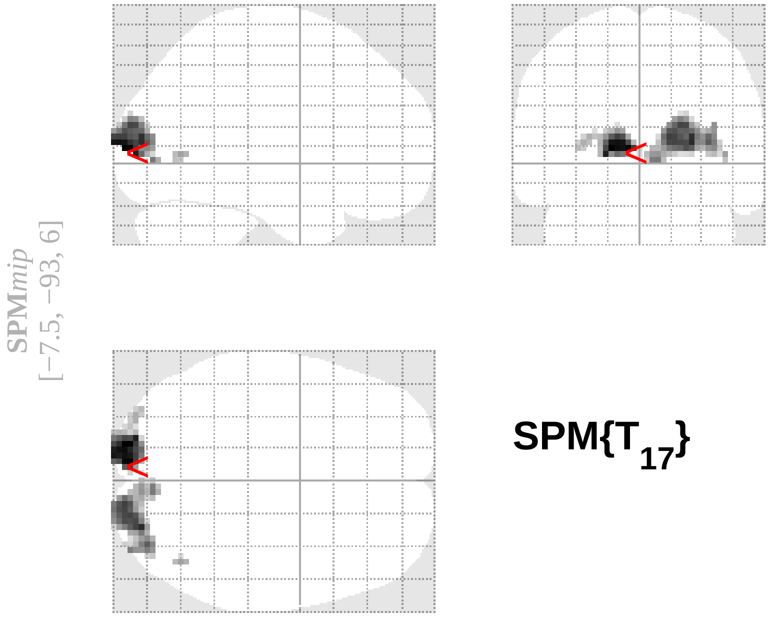

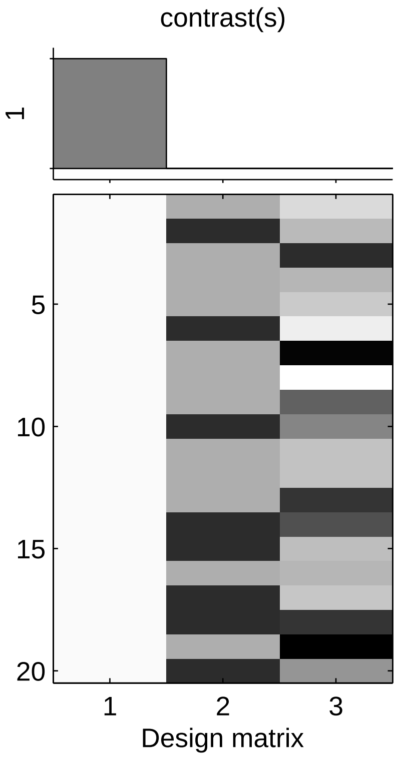

SPM{T_{17}}

SPMresults: .unshifted_pbo_preLD_all_subs
Height threshold T = 3.645767 {p<0.001 (unc.)}
Extent threshold k = 0 voxels

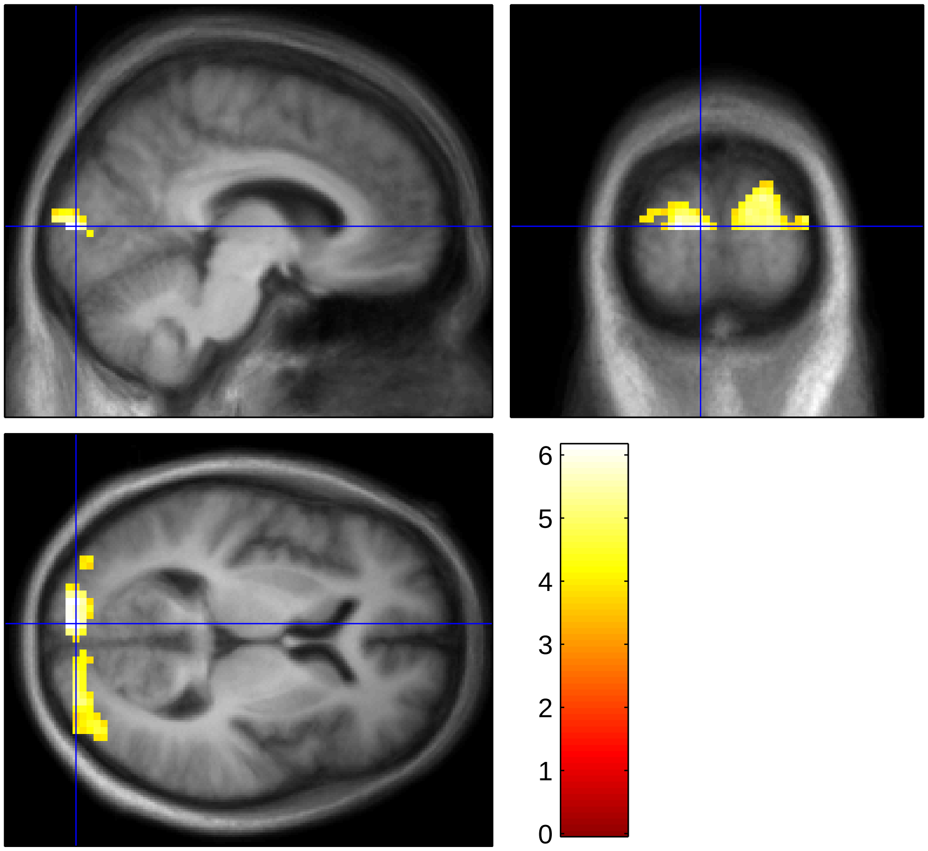

check increases UNSHIFT 20 subs, preLD, pbo day

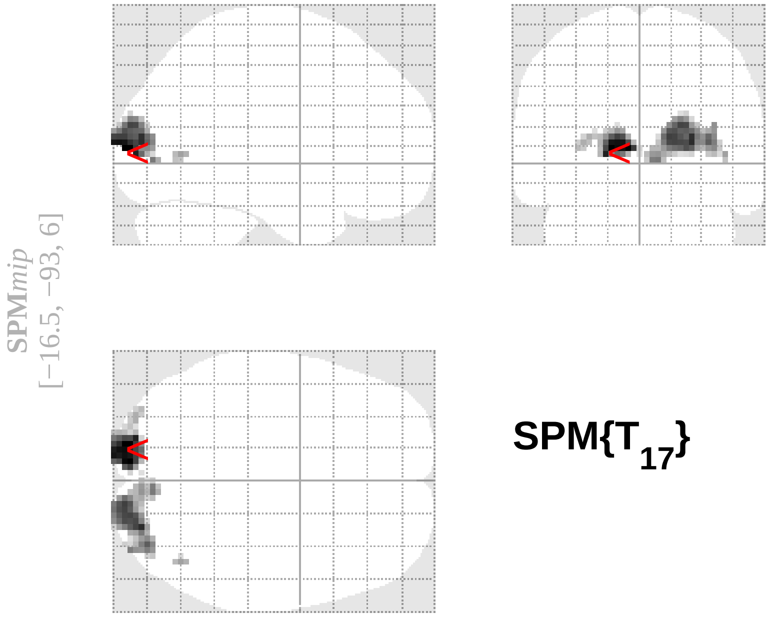

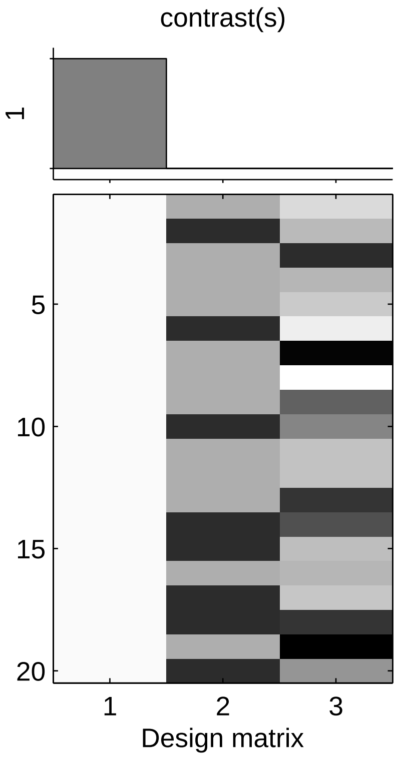

SPMresults: .unshifted_pbo_preLD_all_subs
Height threshold T = 3.645767 {p<0.001 (unc.)}
Extent threshold k = 0 voxels

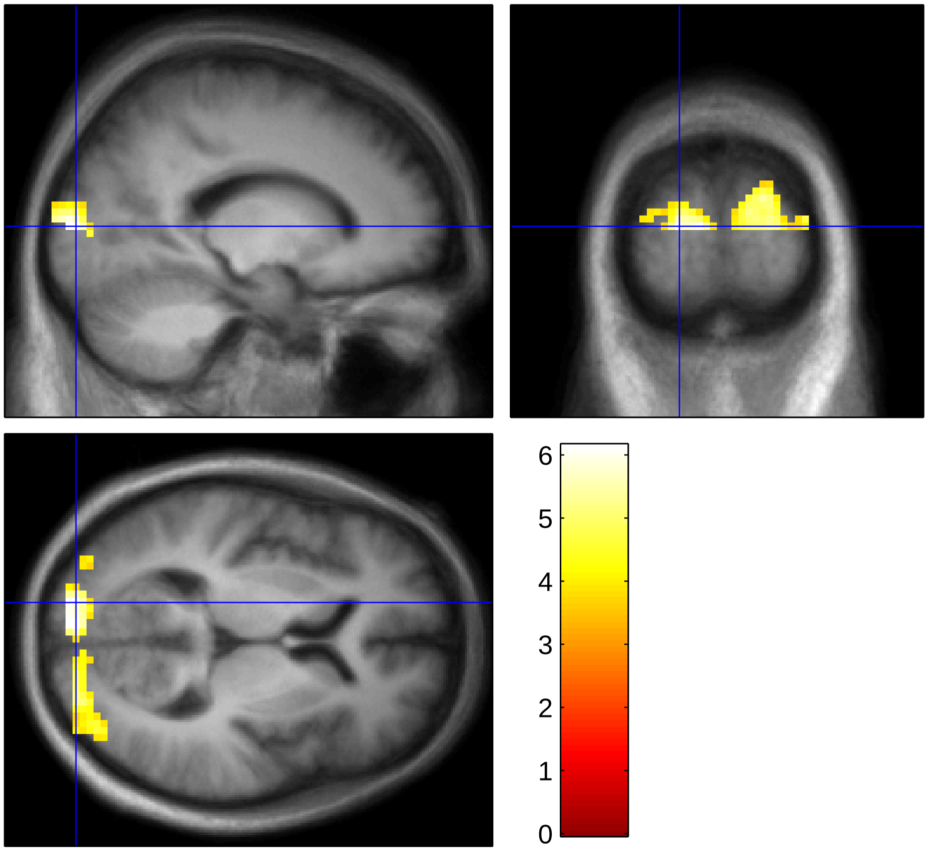

check increases UNSHIFT 20 subs, preLD, pbo day

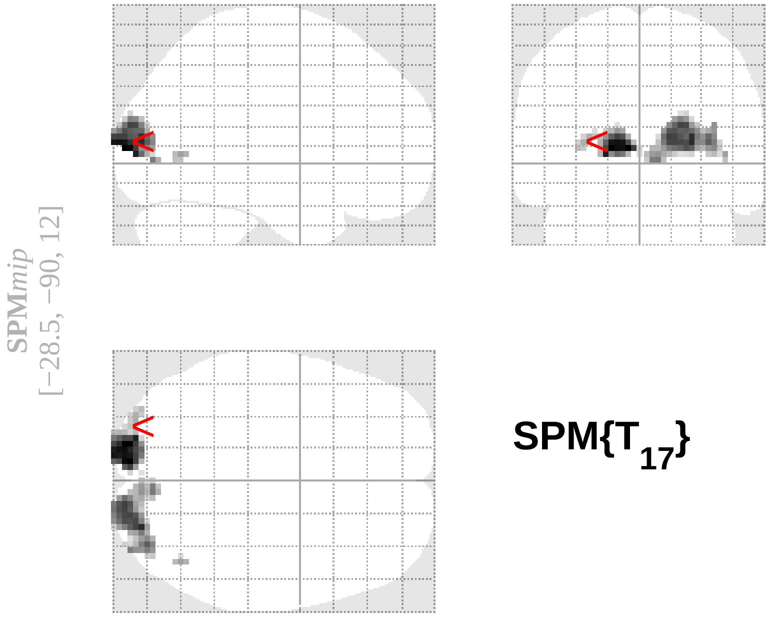

SPM{T_{17}}

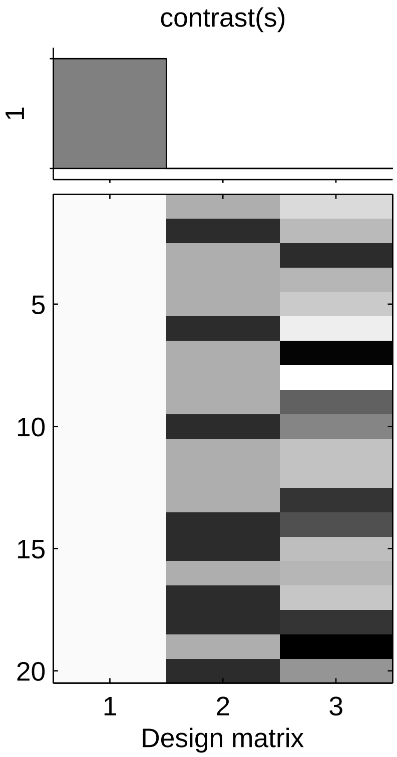

SPMresults: .unshifted_pbo_preLD_all_subs
Height threshold T = 3.645767 {p<0.001 (unc.)}
Extent threshold k = 0 voxels

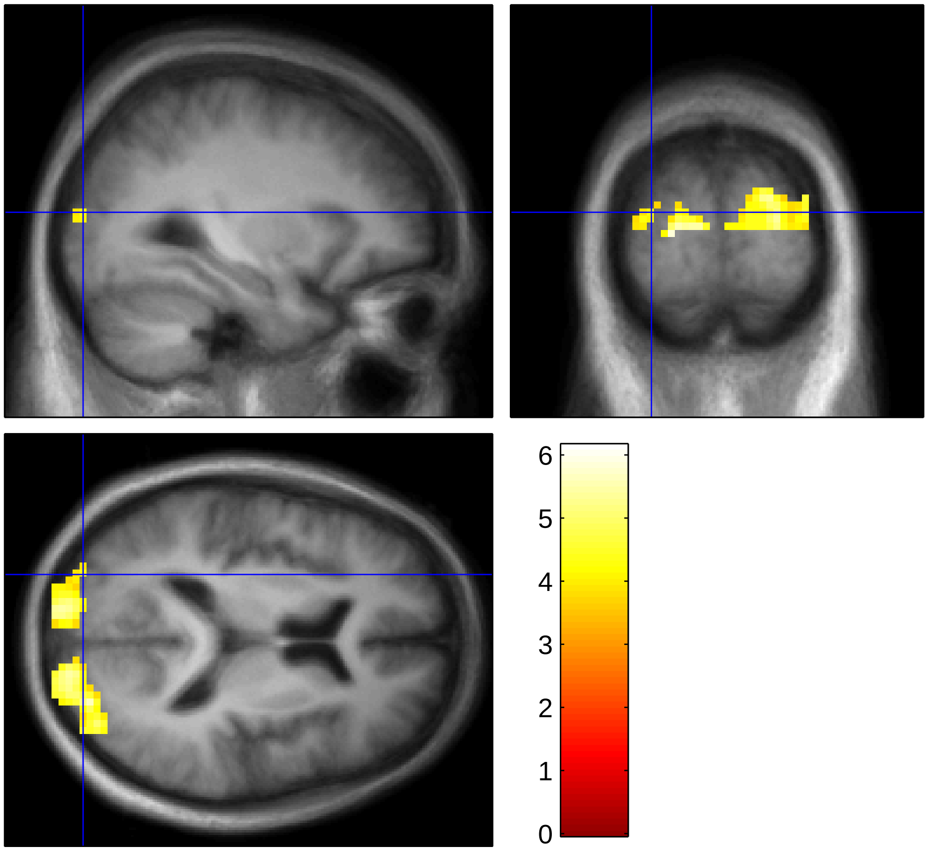

check increases UNSHIFT 20 subs, preLD, pbo day

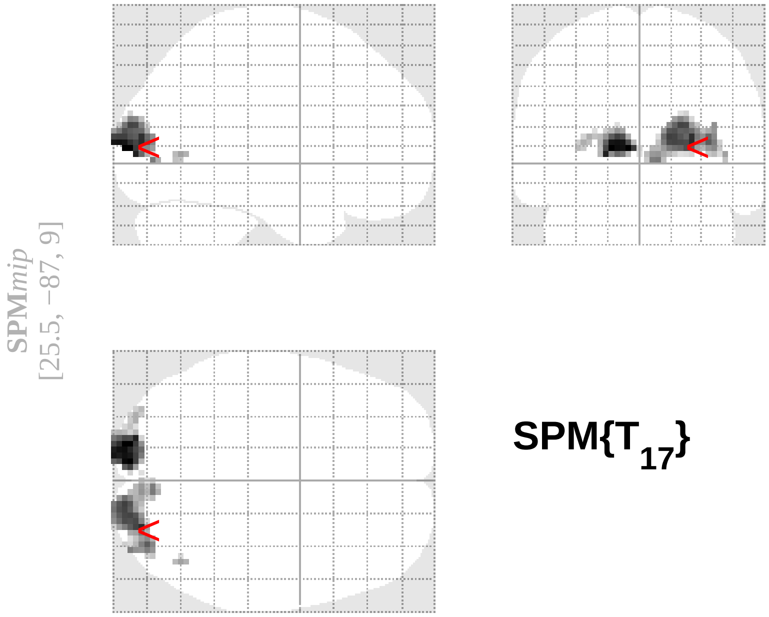

SPM{T_{17}}

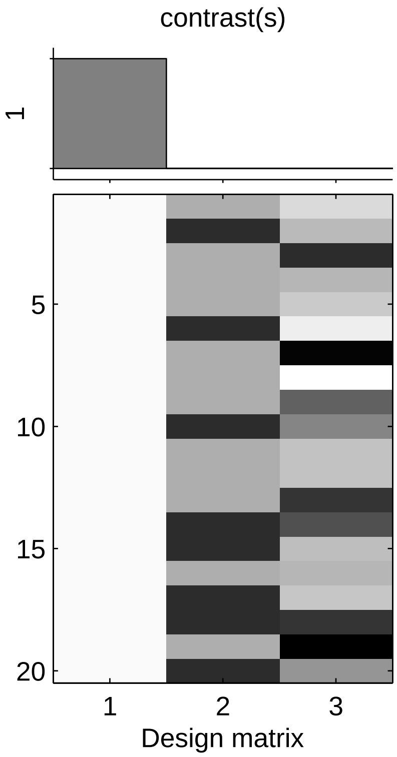

SPMresults: .unshifted_pbo_preLD_all_subs
Height threshold T = 3.645767 {p<0.001 (unc.)}
Extent threshold k = 0 voxels

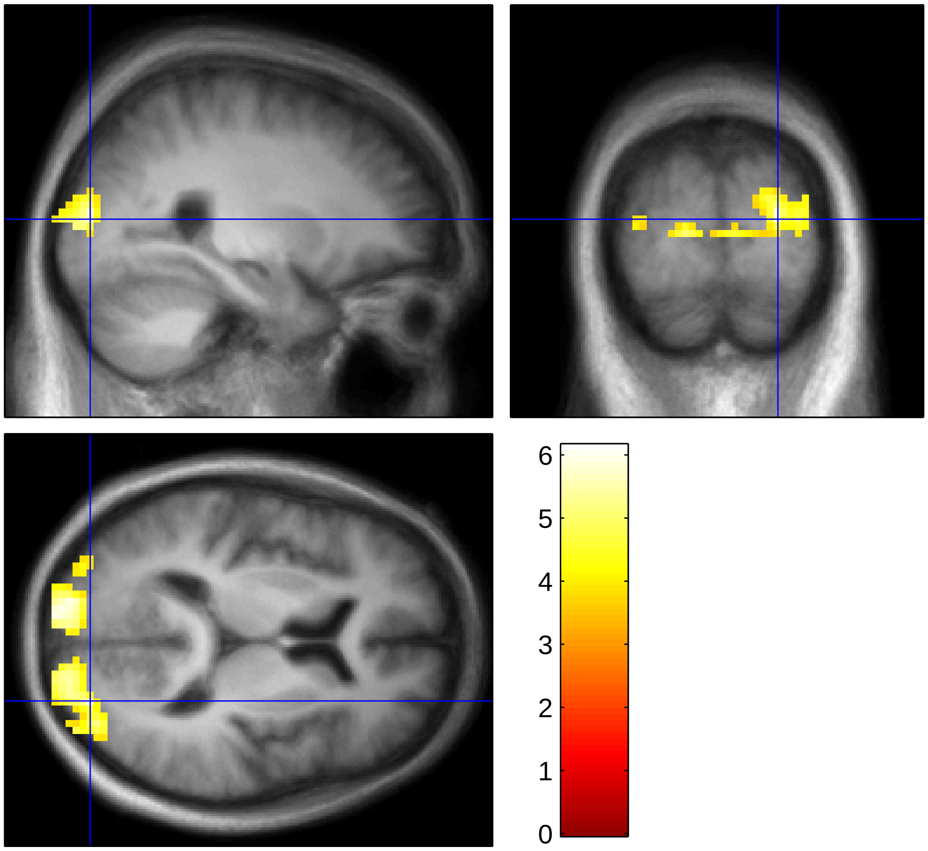

check increases UNSHIFT 20 subs, preLD, pbo day

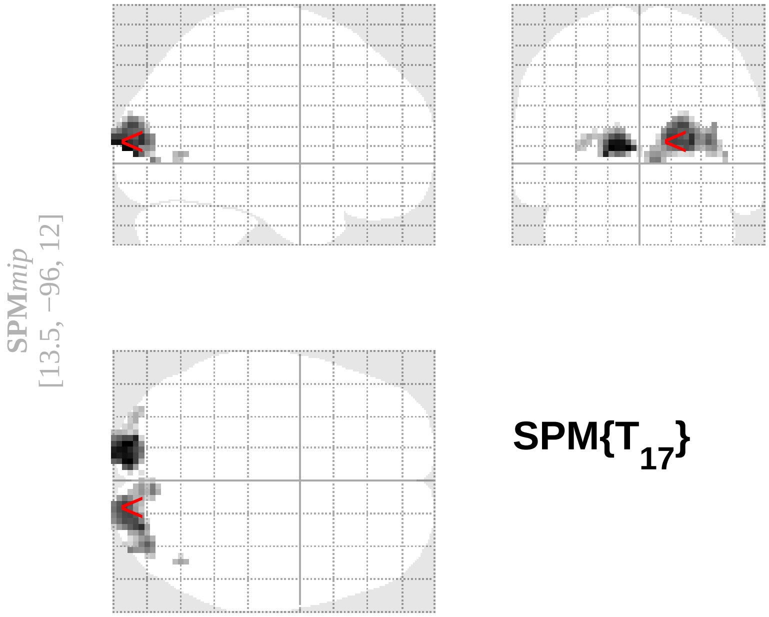

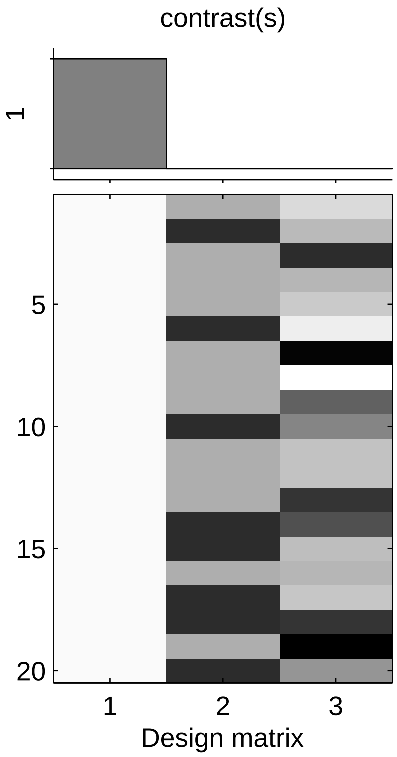

SPM{T_{17}}

SPMresults: .unshifted_pbo_preLD_all_subs
Height threshold T = 3.645767 {p<0.001 (unc.)}
Extent threshold k = 0 voxels

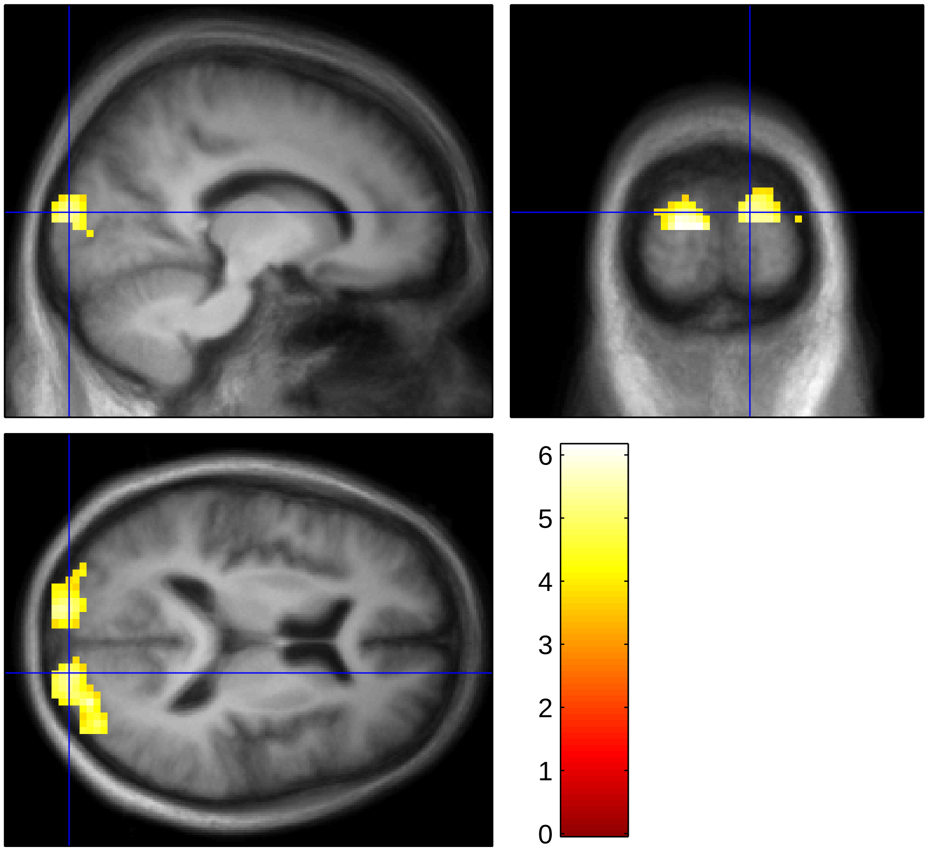

check increases UNSHIFT 20 subs, preLD, pbo day

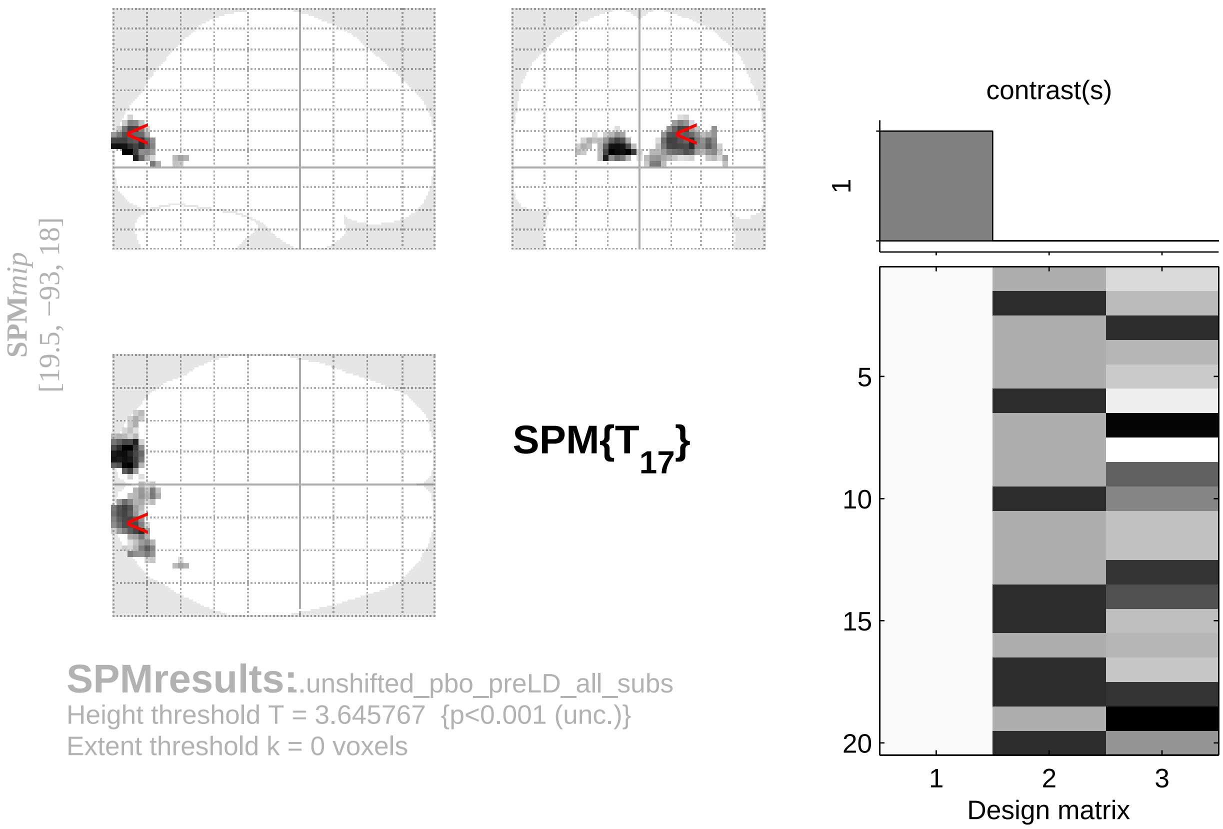

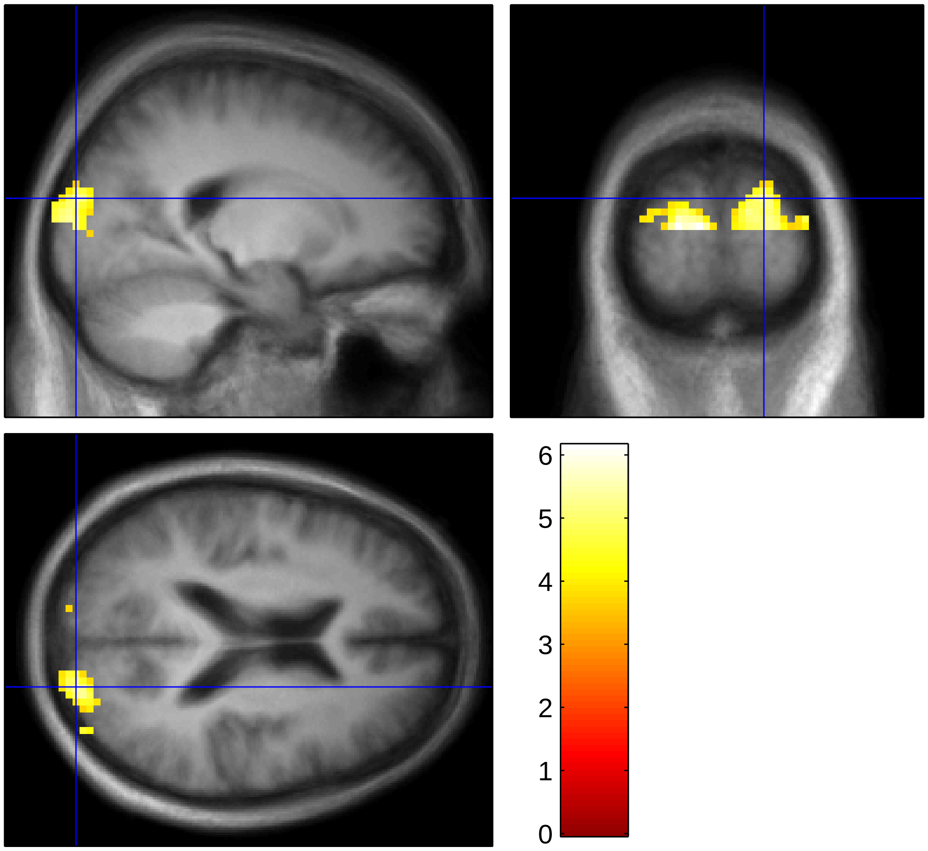

check decreases UNSHIFT 20 subs preLD, pbo only

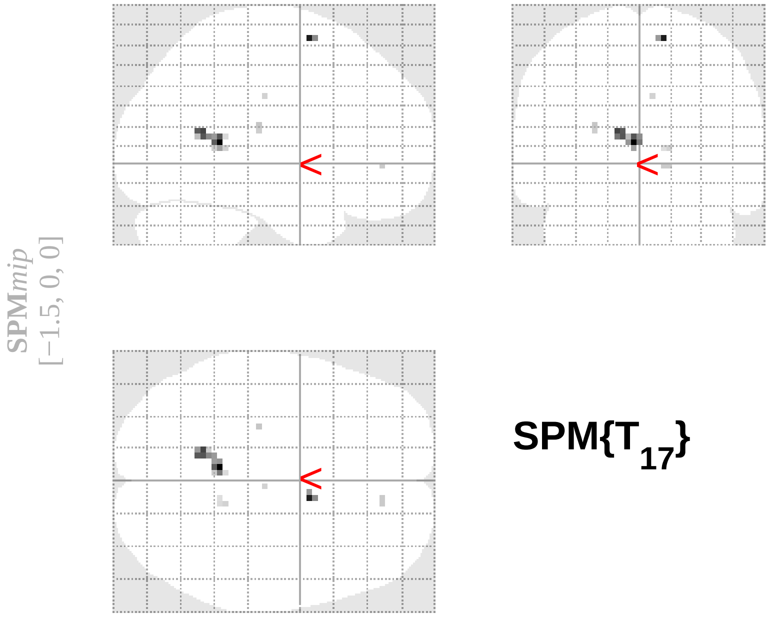

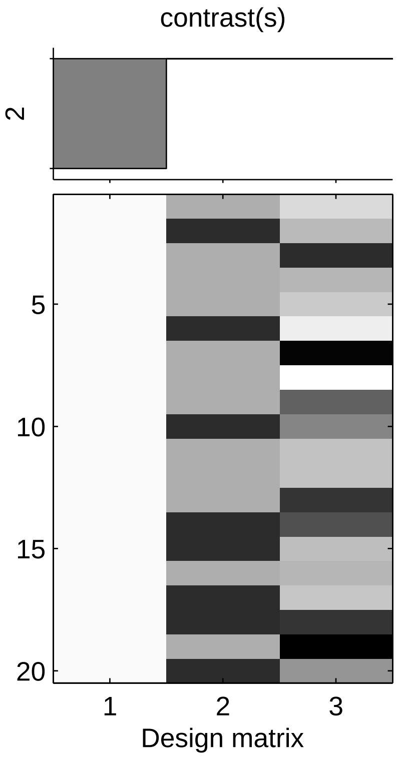

SPMresults: .unshifted_pbo_preLD_all_subs
Height threshold T = 3.645767 {p<0.001 (unc.)}
Extent threshold k = 0 voxels

Statistics: ***p−values adjusted for search volume***

set−level		cluster−level				peak−level					mm mm mm
p	c	$p_{FWE-corr}$	$q_{FDR-corr}$	k_E	p_{uncorr}	$p_{FWE-corr}$	$q_{FDR-corr}$	T	$(Z_{\equiv})$	p_{uncorr}	
**0.809**	**6**	**0.399**	**0.382**	**23**	**0.064**	**0.972**	**0.958**	**4.20**	**3.43**	**0.000**	**-4 -45 9**
						0.991	0.958	4.02	3.32	0.000	-14 -54 15
		**0.981**	**0.699**	**3**	**0.493**	**0.983**	**0.958**	**4.12**	**3.38**	**0.000**	**10 3 63**
		**0.991**	**0.699**	**2**	**0.582**	**0.999**	**0.958**	**3.71**	**3.13**	**0.001**	**-26 -24 18**
		**0.991**	**0.699**	**2**	**0.582**	**0.999**	**0.958**	**3.70**	**3.13**	**0.001**	**10 42 -3**
		**0.997**	**0.711**	**1**	**0.711**	**1.000**	**0.958**	**3.68**	**3.11**	**0.001**	**4 -21 33**
		**0.981**	**0.699**	**3**	**0.493**	**1.000**	**0.958**	**3.68**	**3.11**	**0.001**	**14 -42 6**

table shows 3 local maxima more than 8.0mm apart

Height threshold: T = 3.65, p = 0.001 (1.000)
Extent threshold: k = 0 voxels
Expected voxels per cluster, <k> = 6.691
Expected number of clusters, <c> = 8.00
FWEp: 7.013, FDRp: Inf, FWEc: Inf, FDRc: Inf

Degrees of freedom = [1.0, 17.0]
FWHM = 12.7 13.8 13.7 mm mm mm; 4.2 4.6 4.6 {voxels}
Volume: 1294110 = 47930 voxels = 486.0 resels
Voxel size: 3.0 3.0 3.0 mm mm mm; (resel = 88.62 voxels)
