# Supplementary material for: Additive global cerebral blood flow normalization in arterial spin labeling perfusion imaging
Source: PeerJ. 2015 Mar 17;3:e834. doi: 10.7717/peerj.834 (PMC4369335; doi:10.7717/peerj.834)
Supplement: Supplemental Information 4 — This SPM analysis used images that were multiplicatively normalized for global cerebral blood flow. The first page has the table with the clusters of activation. Pages 2-4 show the the top 3 peaks of activation for the significant cluster of activation. The last page has the table with the clusters of deactivation. There were no significant areas of deactivation. [file peerj-03-834-s004.pdf]

check increases MULT 20 subs, preLD, pbo day only

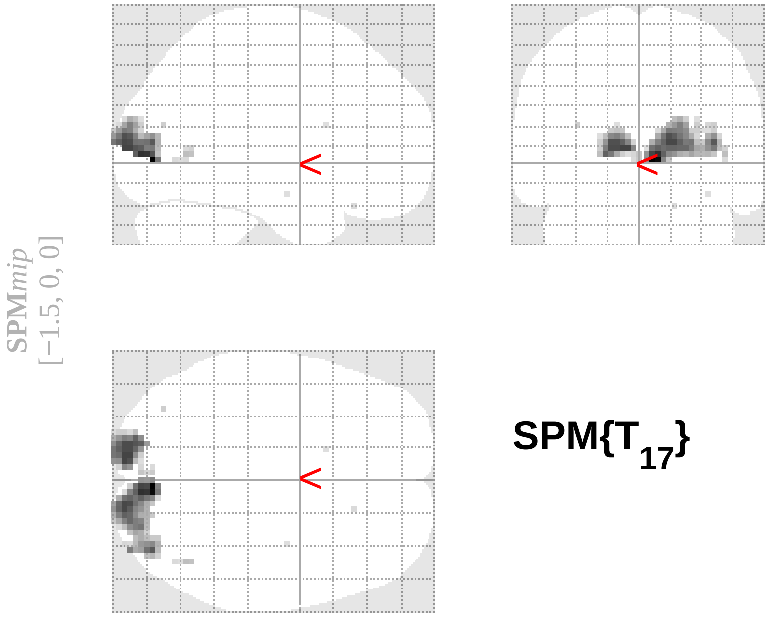

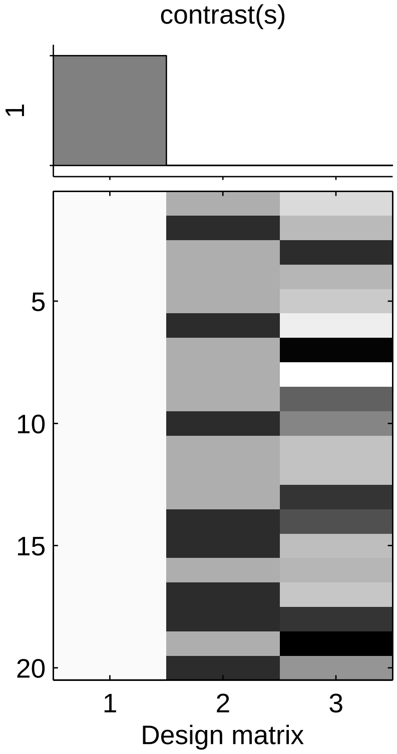

SPMresults: .ects_mult_pbo_preLD_all_subs
Height threshold T = 3.645767 {p<0.001 (unc.)}
Extent threshold k = 0 voxels

Statistics: *p−values adjusted for search volume*

set−level		cluster−level				peak−level					mm mm mm		
p	c	$p_{FWE-corr}$	$q_{FDR-corr}$	k_E	p_{uncorr}	$p_{FWE-corr}$	$q_{FDR-corr}$	T	$(Z_{\equiv})$	p_{uncorr}			
**0.877**	**6**	**0.000**	**0.000**	**369**	**0.000**	**0.027**	**0.063**	**7.36**	**4.87**	**0.000**	**4**	**-81**	**0**
						0.182	0.083	6.19	4.42	0.000	-10	-96	6
						0.199	0.083	6.12	4.39	0.000	14	-96	9
		**0.902**	**0.693**	**7**	**0.261**	**0.980**	**0.663**	**4.21**	**3.44**	**0.000**	**44**	**-63**	**3**
		**0.998**	**0.693**	**1**	**0.693**	**0.997**	**0.891**	**3.95**	**3.28**	**0.001**	**-34**	**-75**	**18**
		**0.998**	**0.693**	**1**	**0.693**	**1.000**	**0.940**	**3.73**	**3.15**	**0.001**	**16**	**27**	**-24**
		**0.998**	**0.693**	**1**	**0.693**	**1.000**	**0.940**	**3.70**	**3.13**	**0.001**	**-14**	**12**	**18**
		**0.998**	**0.693**	**1**	**0.693**	**1.000**	**0.940**	**3.69**	**3.12**	**0.001**	**34**	**-9**	**-18**

table shows 3 local maxima more than 8.0mm apart

Height threshold: T = 3.65, p = 0.001 (1.000)
Extent threshold: k = 0 voxels
Expected voxels per cluster, <k> = 5.987
Expected number of clusters, <c> = 8.88
FWEp: 7.013, FDRp: Inf, FWEc: 369, FDRc: 369

Degrees of freedom = [1.0, 17.0]
FWHM = 12.1 13.4 13.2 mm mm mm; 4.0 4.5 4.4 {voxels}
Volume: 1294110 = 47930 voxels = 543.1 resels
Voxel size: 3.0 3.0 3.0 mm mm mm; (resel = 79.30 voxels)

check increases MULT 20 subs, preLD, pbo day only

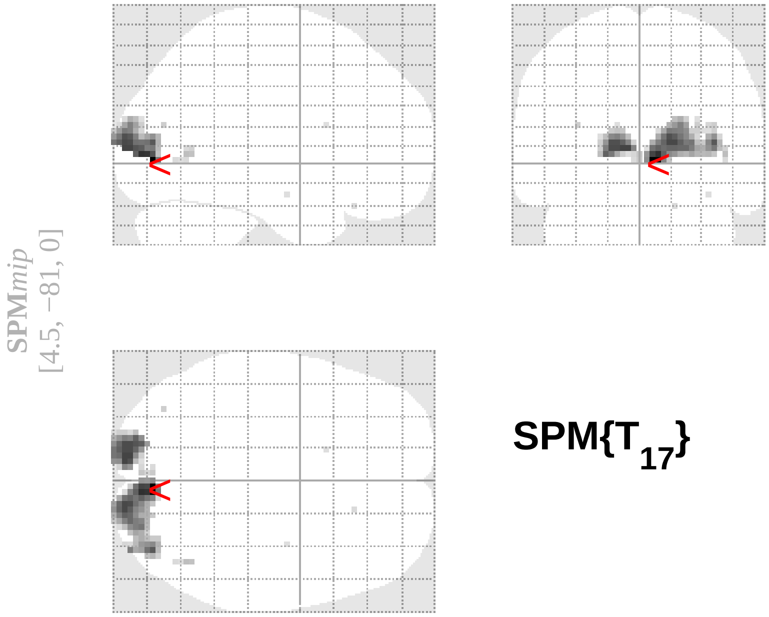

SPM{T_{17}}

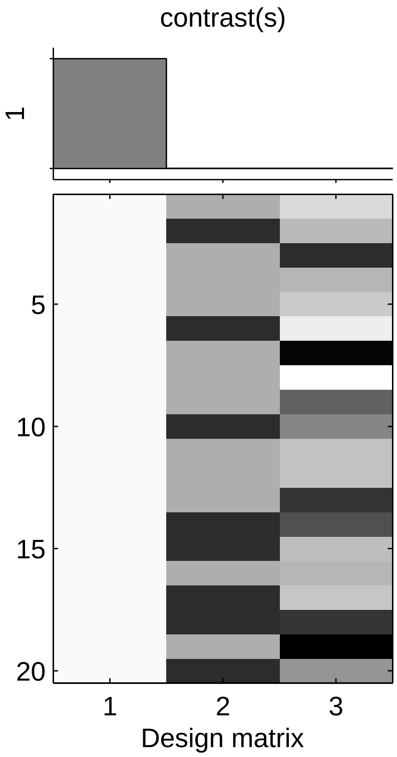

SPMresults:.ects_mult_pbo_preLD_all_subs
Height threshold T = 3.645767 {p<0.001 (unc.)}
Extent threshold k = 0 voxels

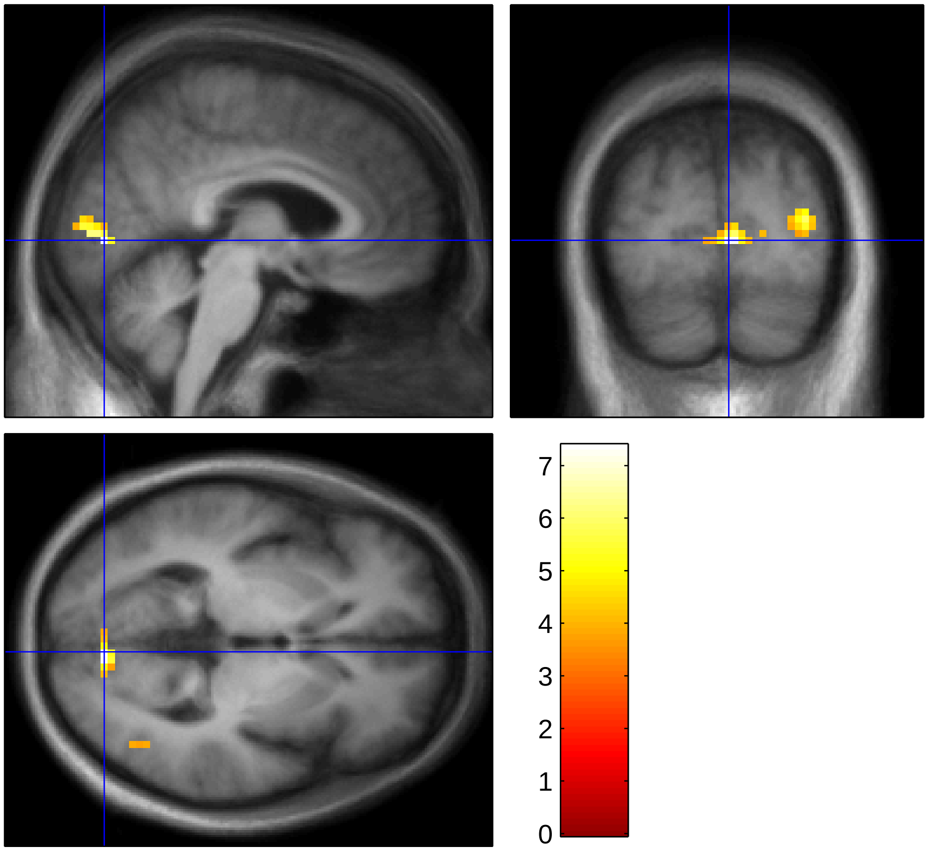

check increases MULT 20 subs, preLD, pbo day only

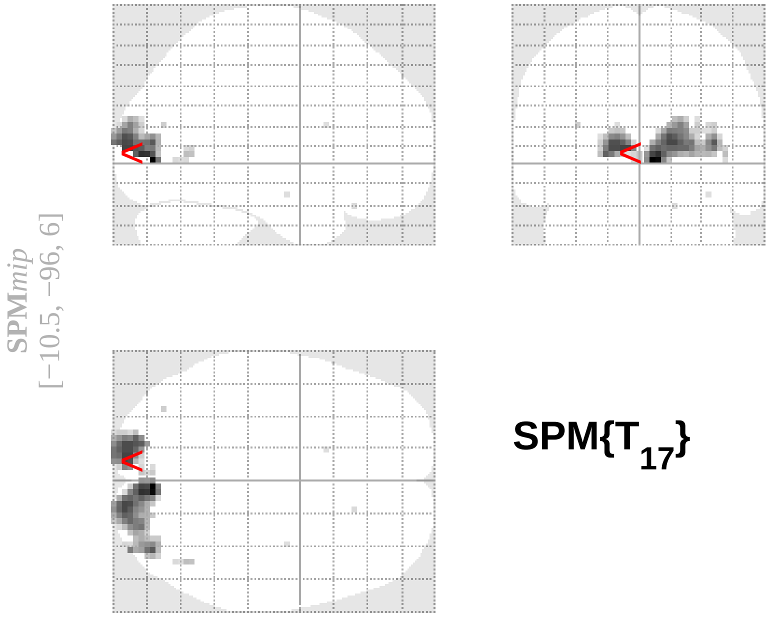

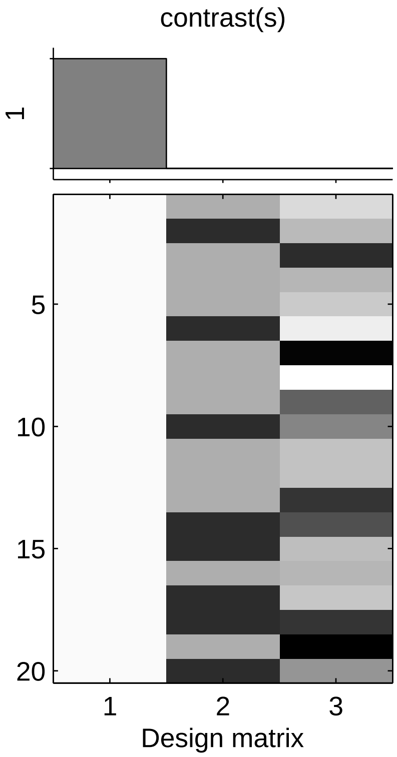

SPMresults: .ects_mult_pbo_preLD_all_subs
Height threshold T = 3.645767 {p<0.001 (unc.)}
Extent threshold k = 0 voxels

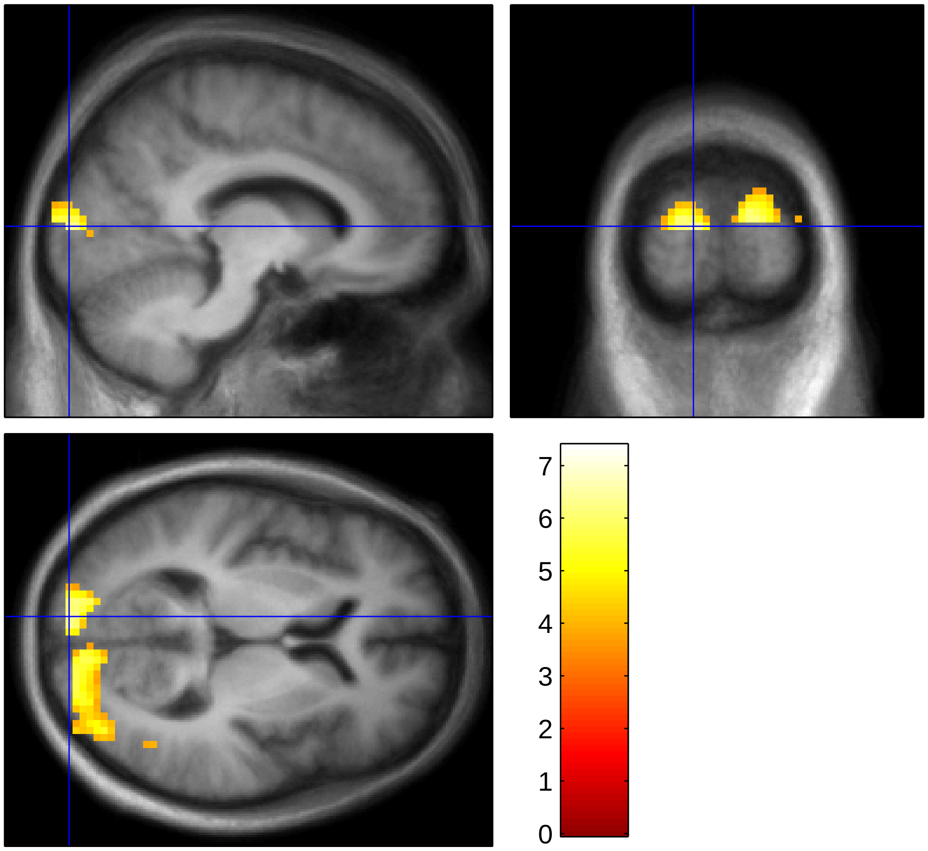

check increases MULT 20 subs, preLD, pbo day only

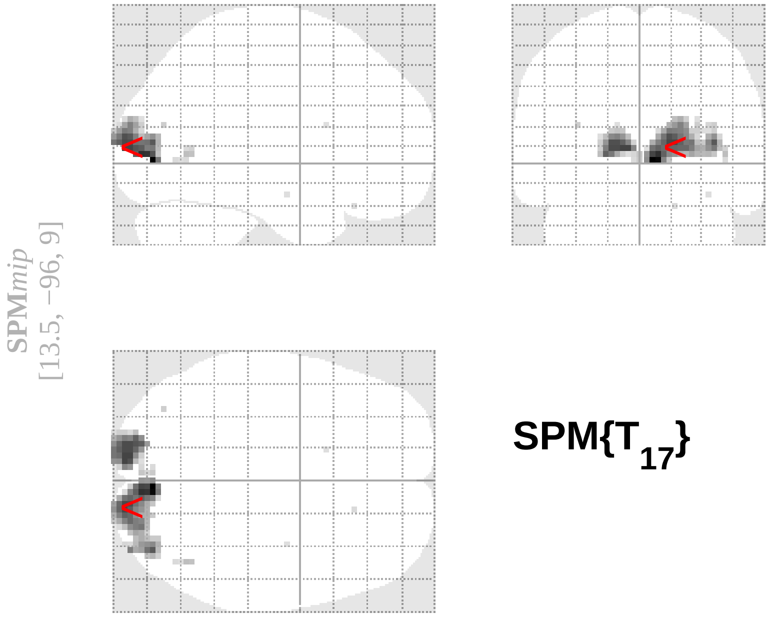

SPM{T_{17}}

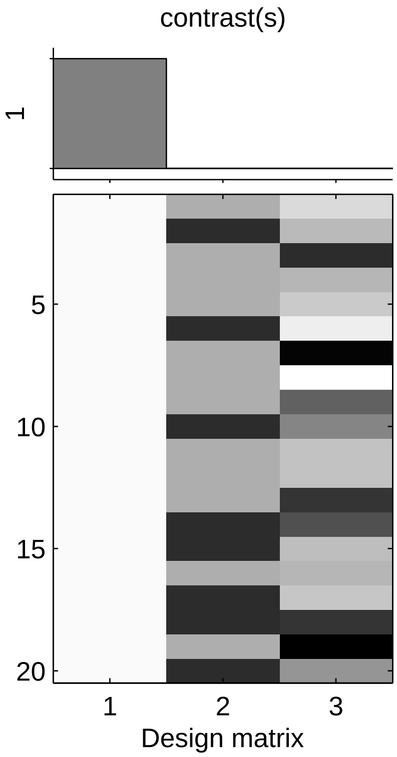

SPMresults: .ects_mult_pbo_preLD_all_subs
Height threshold T = 3.645767 {p<0.001 (unc.)}
Extent threshold k = 0 voxels

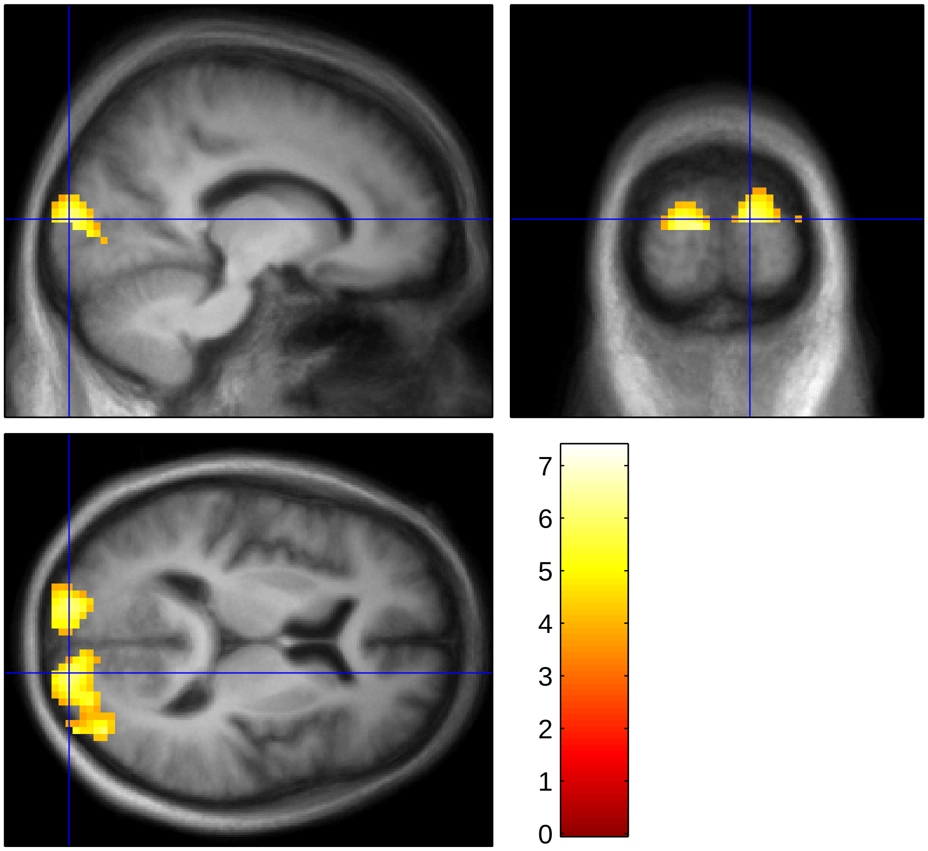

check decreases MULT 20 subs preLD, pbo day only

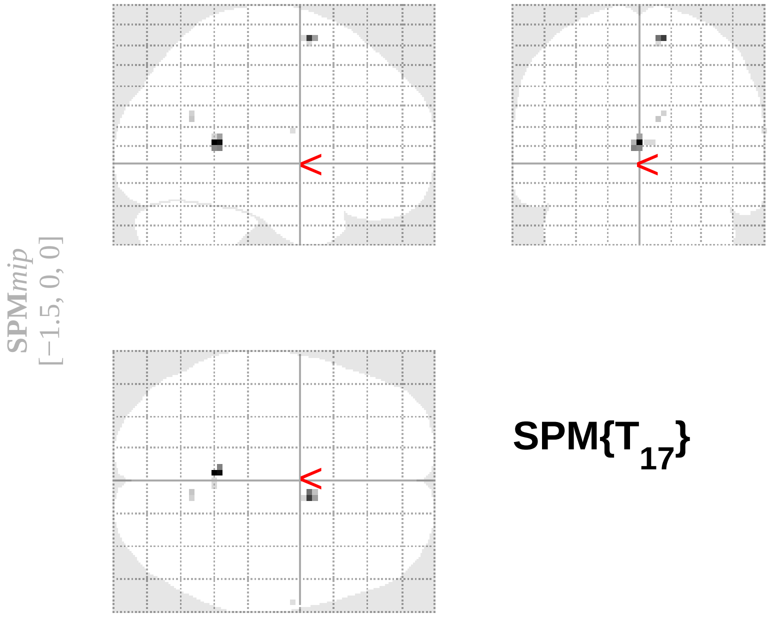

SPM{T_{17}}

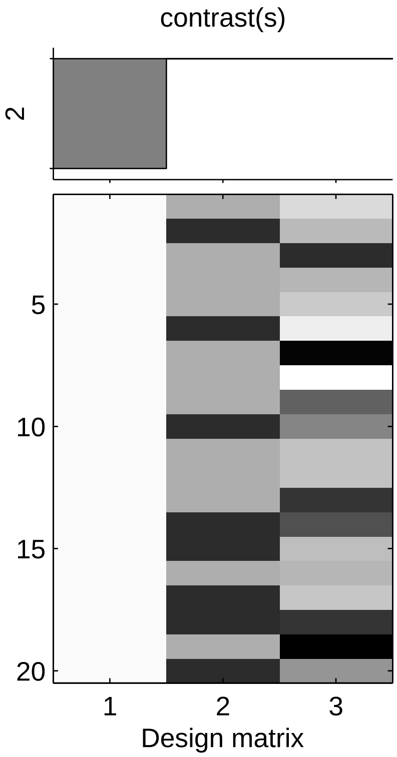

SPMresults:.ects_mult_pbo_preLD_all_subs
Height threshold T = 3.645767 {p<0.001 (unc.)}
Extent threshold k = 0 voxels

Statistics: ***p−values adjusted for search volume***

set−level		cluster−level				peak−level					mm mm mm
p	c	$p_{FWE-corr}$	$q_{FDR-corr}$	k_E	p_{uncorr}	$p_{FWE-corr}$	$q_{FDR-corr}$	T	$(Z_\equiv)$	p_{uncorr}	
0.977	4	0.802	0.596	10	0.182	0.917	0.983	4.52	3.61	0.000	−2 −48 9
		0.929	0.596	6	0.298	0.972	0.983	4.27	3.47	0.000	10 3 63
		0.993	0.693	2	0.559	1.000	0.983	3.75	3.16	0.001	8 −60 21
		0.998	0.693	1	0.693	1.000	0.983	3.66	3.10	0.001	64 −6 15

table shows 3 local maxima more than 8.0mm apart

Height threshold: T = 3.65, p = 0.001 (1.000)
Extent threshold: k = 0 voxels
Expected voxels per cluster, <k> = 5.987
Expected number of clusters, <c> = 8.88
FWEp: 7.013, FDRp: Inf, FWEc: Inf, FDRc: Inf

Degrees of freedom = [1.0, 17.0]
FWHM = 12.1 13.4 13.2 mm mm mm; 4.0 4.5 4.4 {voxels}
Volume: 1294110 = 47930 voxels = 543.1 resels
Voxel size: 3.0 3.0 3.0 mm mm mm; (resel = 79.30 voxels)
