# Supplementary material for: Additive global cerebral blood flow normalization in arterial spin labeling perfusion imaging
Source: PeerJ. 2015 Mar 17;3:e834. doi: 10.7717/peerj.834 (PMC4369335; doi:10.7717/peerj.834)
Supplement: Supplemental Information 5 — This SPM analysis used images that were additively normalized for global cerebral blood flow. The first page has the table with the clusters of activation. Pages 2–4 show the the top 3 peaks of activation for the significant cluster of activation. The last page has the table with the clusters of deactivation. There were no significant areas of deactivation. [file peerj-03-834-s005.pdf]

check increases SHIFT 20 subs preLD, pbo day

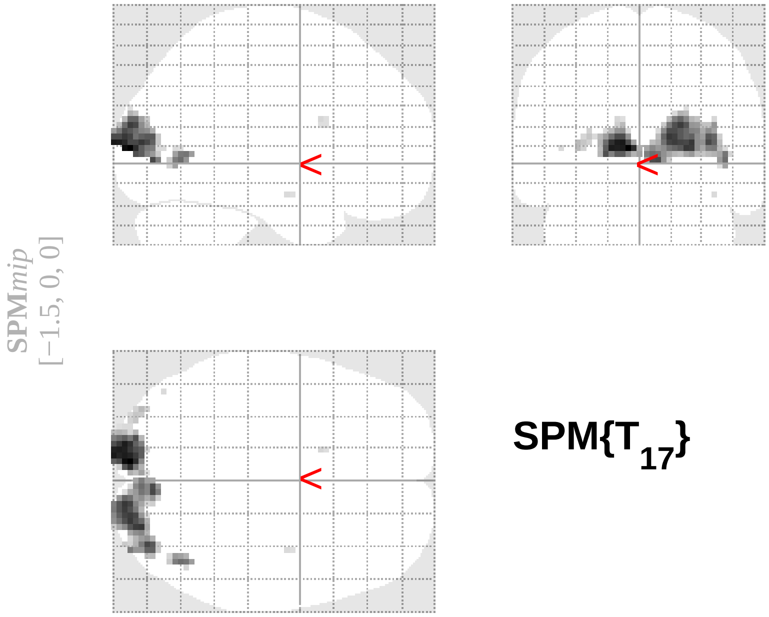

SPM$\{T_{17}\}$

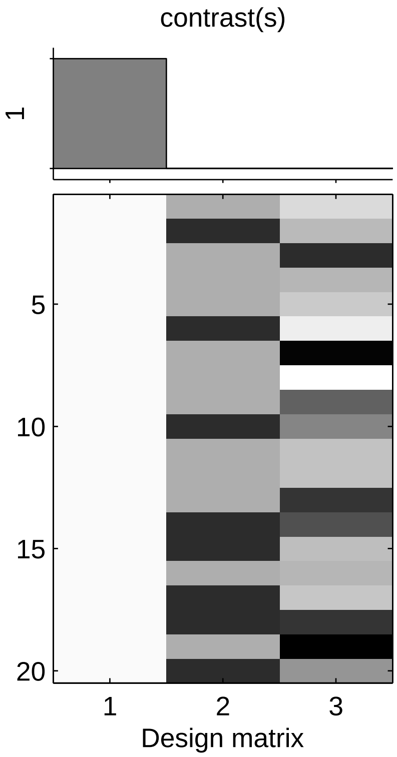

SPMresults:.s_shifted_pbo_preLD_all_subs
Height threshold T = 3.645767 {p<0.001 (unc.)}
Extent threshold k = 0 voxels

Statistics: ***p−values adjusted for search volume***

set−level		cluster−level				peak−level					mm mm mm		
p	c	$p_{FWE-corr}$	$q_{FDR-corr}$	k_E	p_{uncorr}	$p_{FWE-corr}$	$q_{FDR-corr}$	T	$(Z_{\equiv})$	p_{uncorr}			
**0.961**	**5**	**0.000**	**0.000**	**447**	**0.000**	**0.131**	**0.129**	**6.48**	**4.54**	**0.000**	**−8**	**−93**	**6**
						0.217	0.129	6.11	4.38	0.000	−10	−102	9
						0.233	0.129	6.05	4.36	0.000	−16	−96	6
		**0.396**	**0.132**	**21**	**0.053**	**0.691**	**0.184**	**5.06**	**3.90**	**0.000**	**44**	**−66**	**0**
		**0.973**	**0.629**	**4**	**0.377**	**1.000**	**0.916**	**3.76**	**3.16**	**0.001**	**−14**	**9**	**21**
		**0.994**	**0.677**	**2**	**0.541**	**1.000**	**0.916**	**3.73**	**3.14**	**0.001**	**38**	**−9**	**−18**
		**0.998**	**0.679**	**1**	**0.679**	**1.000**	**0.916**	**3.71**	**3.13**	**0.001**	**−44**	**−75**	**6**

table shows 3 local maxima more than 8.0mm apart

Height threshold: T = 3.65, p = 0.001 (1.000)
Extent threshold: k = 0 voxels
Expected voxels per cluster, <k> = 5.528
Expected number of clusters, <c> = 9.56
FWEp: 7.013, FDRp: Inf, FWEc: 447, FDRc: 447

Degrees of freedom = [1.0, 17.0]
FWHM = 11.9 13.0 12.8 mm mm mm; 4.0 4.3 4.3 {voxels}
Volume: 1294110 = 47930 voxels = 588.2 resels
Voxel size: 3.0 3.0 3.0 mm mm mm; (resel = 73.21 voxels)

check increases SHIFT 20 subs preLD, pbo day

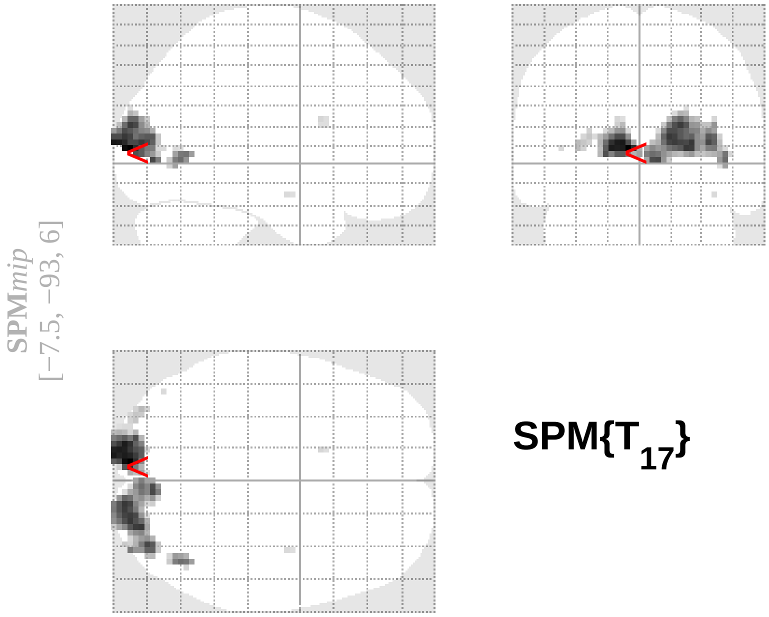

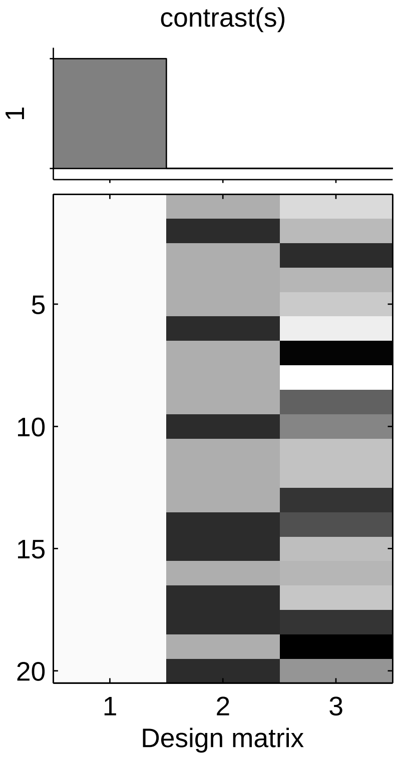

SPMresults:.s_shifted_pbo_preLD_all_subs
Height threshold T = 3.645767 {p<0.001 (unc.)}
Extent threshold k = 0 voxels

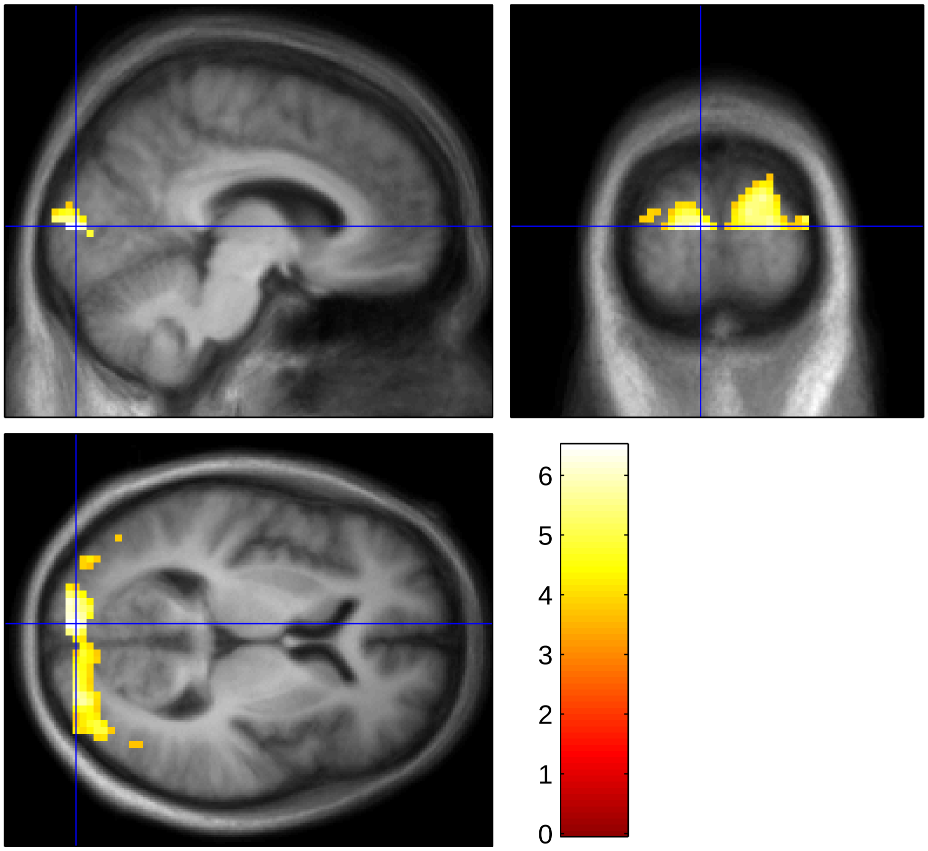

check increases SHIFT 20 subs preLD, pbo day

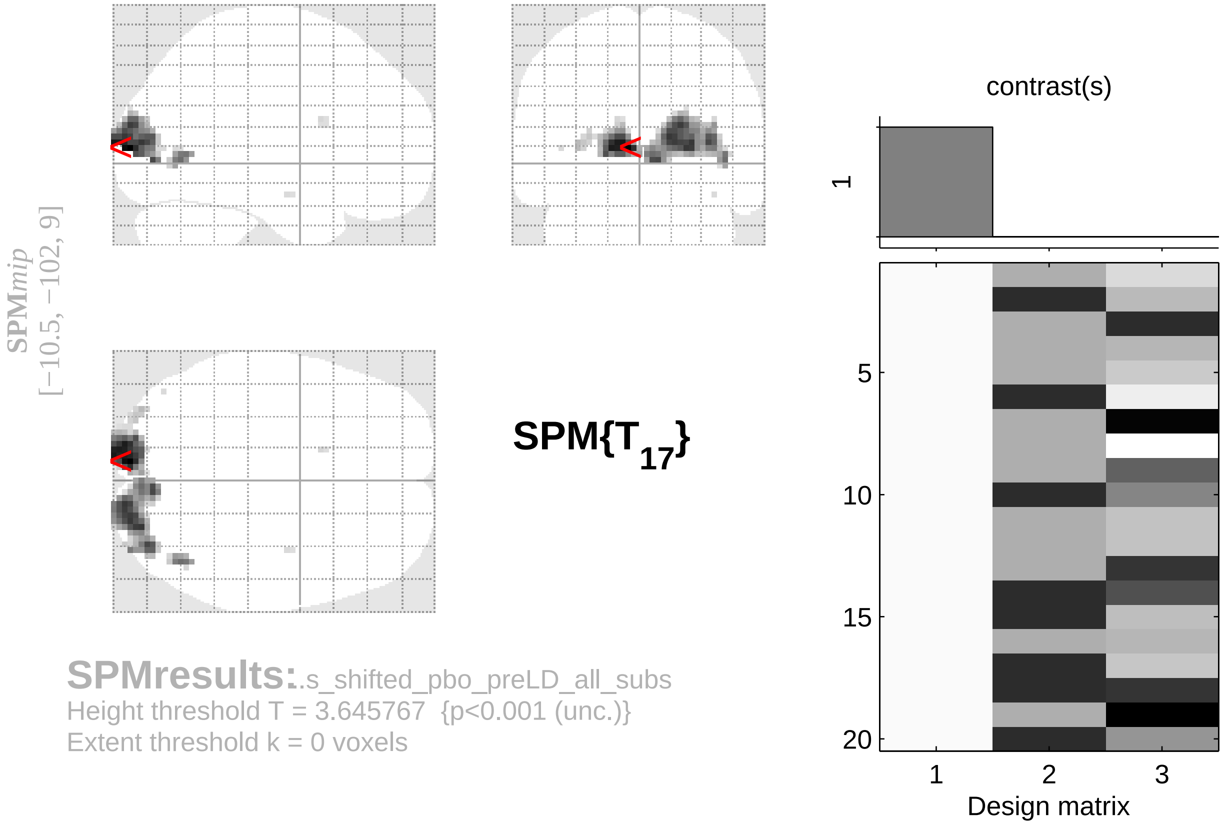

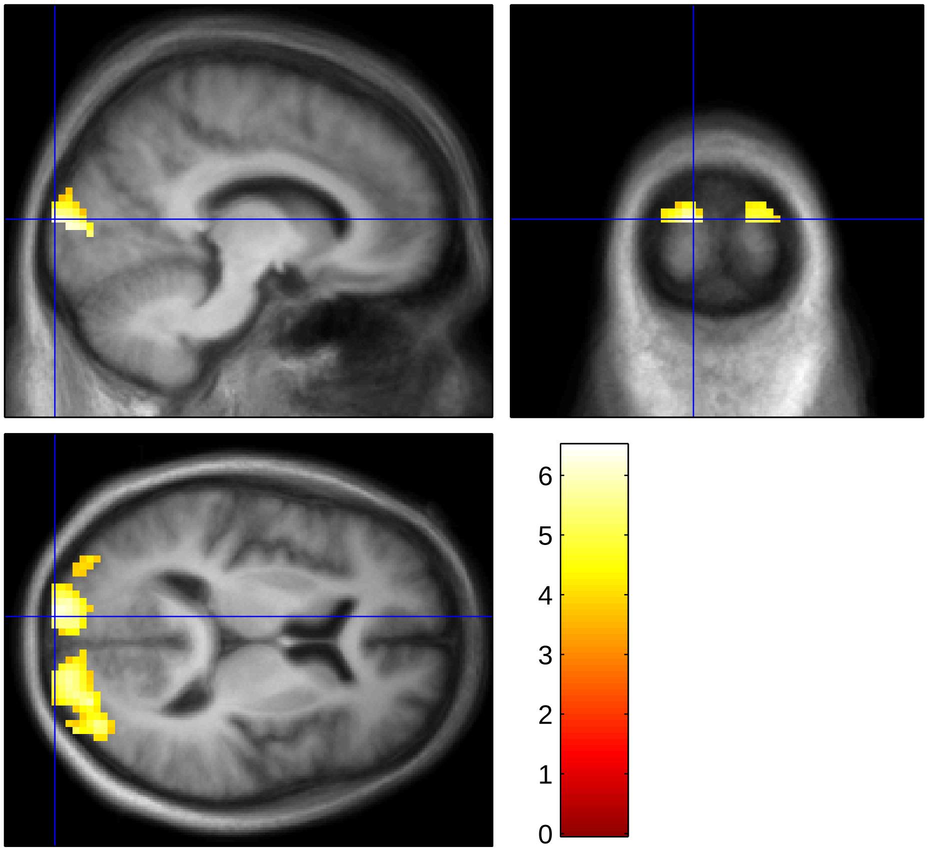

check increases SHIFT 20 subs preLD, pbo day

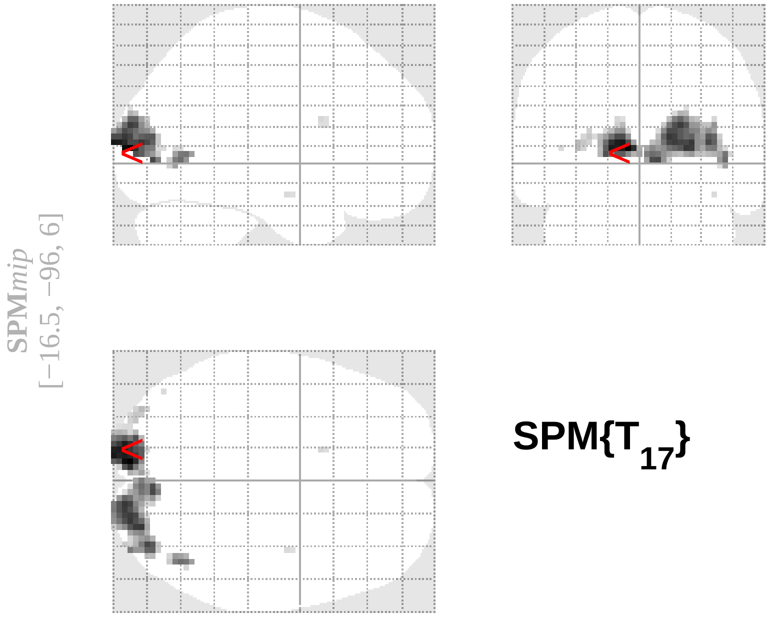

$SPM\{T_{17}\}$

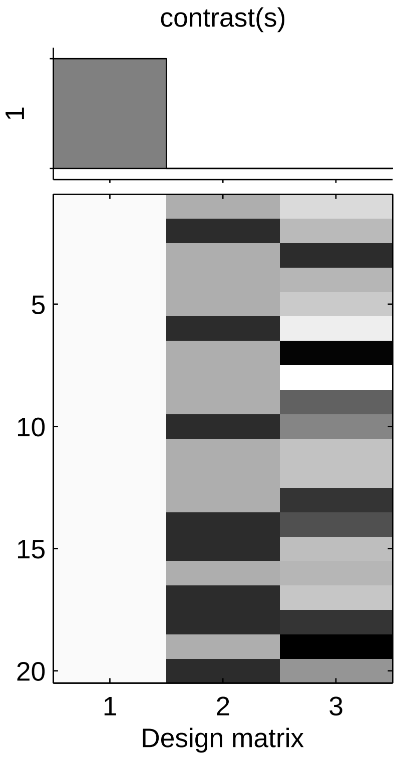

SPMresults:.s_shifted_pbo_preLD_all_subs
Height threshold T = 3.645767 {p<0.001 (unc.)}
Extent threshold k = 0 voxels

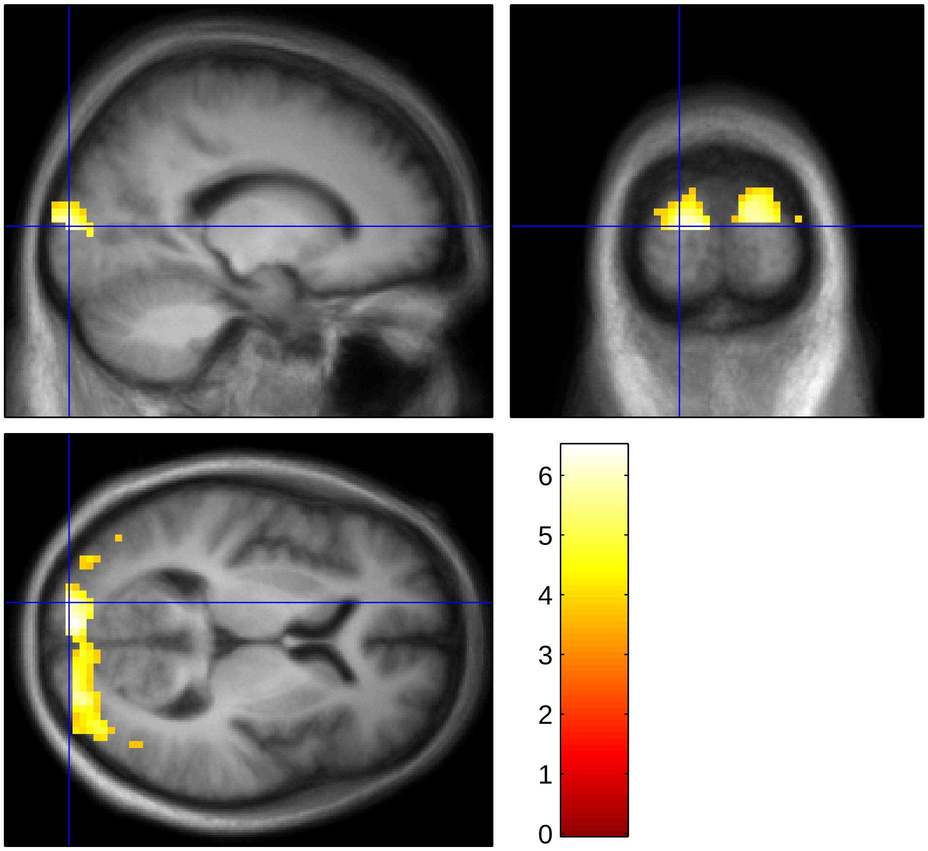

check decreases SHIFT 20 subs preLD, pbo day

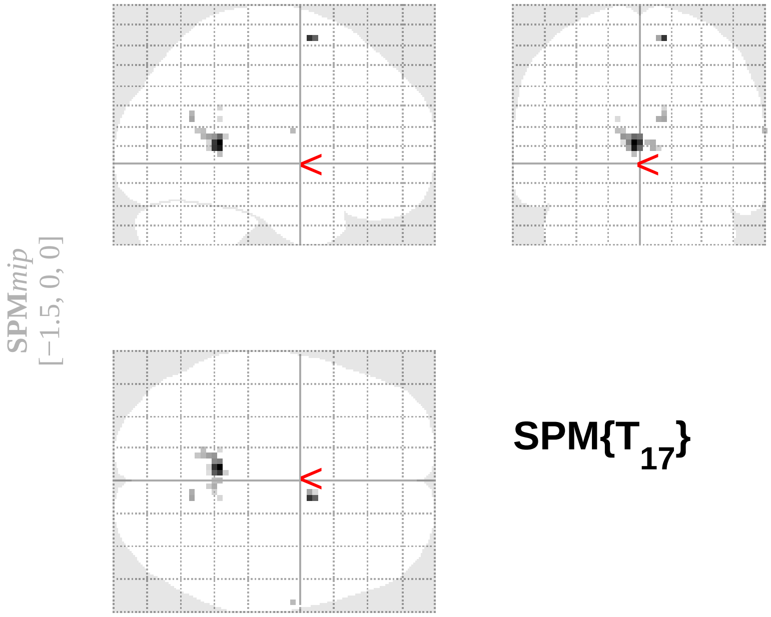

SPM{T_{17}}

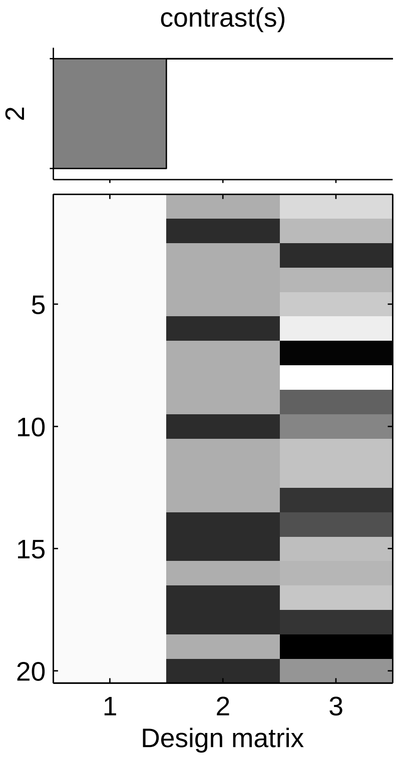

SPMresults:.s_shifted_pbo_preLD_all_subs
Height threshold T = 3.645767 {p<0.001 (unc.)}
Extent threshold k = 0 voxels

Statistics: *p−values adjusted for search volume*

set−level		cluster−level				peak−level					
p	c	$p_{FWE-corr}$	$q_{FDR-corr}$	k_E	p_{uncorr}	$p_{FWE-corr}$	$q_{FDR-corr}$	T	$(Z_{\equiv})$	p_{uncorr}	mm mm mm
**0.914**	**6**	**0.142**	**0.096**	**35**	**0.016**	**0.817**	**0.954**	**4.82**	**3.77**	**0.000**	**-4 -45 9**
						0.999	0.954	3.91	3.25	0.001	4 -48 9
		**0.973**	**0.679**	**4**	**0.377**	**0.928**	**0.954**	**4.53**	**3.62**	**0.000**	**10 3 63**
		**0.986**	**0.679**	**3**	**0.447**	**0.998**	**0.954**	**3.94**	**3.27**	**0.001**	**10 -60 21**
		**0.998**	**0.679**	**1**	**0.679**	**0.999**	**0.954**	**3.84**	**3.21**	**0.001**	**64 -6 15**
		**0.998**	**0.679**	**1**	**0.679**	**1.000**	**0.954**	**3.70**	**3.12**	**0.001**	**10 -45 27**
		**0.998**	**0.679**	**1**	**0.679**	**1.000**	**0.954**	**3.68**	**3.11**	**0.001**	**-14 -45 21**

table shows 3 local maxima more than 8.0mm apart

Height threshold: T = 3.65, p = 0.001 (1.000)
Extent threshold: k = 0 voxels
Expected voxels per cluster, <k> = 5.528
Expected number of clusters, <c> = 9.56
FWEp: 7.013, FDRp: Inf, FWEc: Inf, FDRc: Inf

Degrees of freedom = [1.0, 17.0]
FWHM = 11.9 13.0 12.8 mm mm mm; 4.0 4.3 4.3 {voxels}
Volume: 1294110 = 47930 voxels = 588.2 resels
Voxel size: 3.0 3.0 3.0 mm mm mm; (resel = 73.21 voxels)
